# Norethisterone enanthate-induced cerebral venous sinus thrombosis (CVST)

**DOI:** 10.1136/bcr-2017-222418

**Published:** 2017-11-14

**Authors:** Mandreker Bahall, Manisha Santlal

**Affiliations:** 1 Department of Clinical and Medical Sciences, The University of The West Indies at Saint Augustine Faculty of Medical Sciences, St Augustine, xxxx, Trinidad and Tobago; 2 Department of Medicine, South West Regional Health Authority, San Fernando, Trinidad and Tobago

**Keywords:** drugs and medicines, neurology (drugs and medicines)

## Abstract

A 23-year-old East Indian woman with no significant medical history, except a depot-norethisterone enanthate injection taken 3 weeks prior to admission, presented with a gradually worsening headache for the past 5 days. She had no fever, vomiting, neck stiffness, focal weakness or rash, and examination was unremarkable with no focal neurological deficits. Vasculitic, thrombophilia and sepsis screens were normal. A brain CT scan showed a left parietal lobe venous infarct, secondary to a venous dural sinus thrombosis, with MRI and Magnetic Resonance Venogram (MRV) confirming a signal void. She was diagnosed to have multiple cerebral venous sinus thrombosis due to norethisterone enanthate. She made a complete recovery following treatment with mannitol, dexamethasone and anticoagulants. A follow-up brain MRI done at 6 months was normal.

## Background

Cerebral venous sinus thrombosis (CVST) is a rare condition with only two to five cases per million people per year.^1^ In Trinidad and Tobago, with a population of 1.3 million, few cases are expected. Furthermore, the association between depot-norethisterone enanthate, a progestin-only contraceptive injection and CVST has rarely been reported in the literature. Ramya *et al* and Rajput *et al* reported that norethisterone and norethindrone acetate pills caused CVST in a patient with hyperhomocysteinaemia.^2 3^ This case report highlights the importance of having a high degree of suspicion of CVST in individuals with long-term depot progesterones, and aggressive treatment for optimum and satisfactory outcomes of CVST.

## Case presentation

A 23-year-old married housewife, with two children, presented with a mild headache that gradually worsened over a period of 2 days. Thereafter, she sought emergency medical attention at a District Health Facility where she was treated with analgesia and discharged the same day. However, she had no significant relief in her headache. Five days after the initial onset of her symptoms, the headache worsened, and was associated with multiple episodes of vomiting and one episode of syncope lasting 2–3 min. She experienced no fever, blurred vision, photophobia, gait disturbance, seizure activity or focal sensory and motor deficit. She had no history of head trauma or neck stiffness. She had not taken any over-the-counter medication or prescription medicines such as antipsychotics or herbal medications, except norethisterone enanthate depot injections, which she received every 2 months for 2 years as a contraception following the birth of her last child. Her last dose was 3 weeks prior to the commencement of her symptoms. She was neither diabetic, hypertensive nor had hypercholesterolaemia. Additionally, she had no significant family history, did not smoke and only had alcohol occasionally.

Examination on admission revealed a listless, exhausted patient with a Glasgow Coma Scale score of 15/15. She was oriented in time, place and person. Her blood pressure was 107/72 mm Hg, and pulse was 80 beats per minute and regular. Central nervous system examination revealed no abnormalities. Rest of the neurological examination, including cranial nerves, sensory and motor systems, deep tendon reflexes and planters were normal. Kernig’s and Brudzinski’s signs were negative.

## Investigations

**Figure 1:**
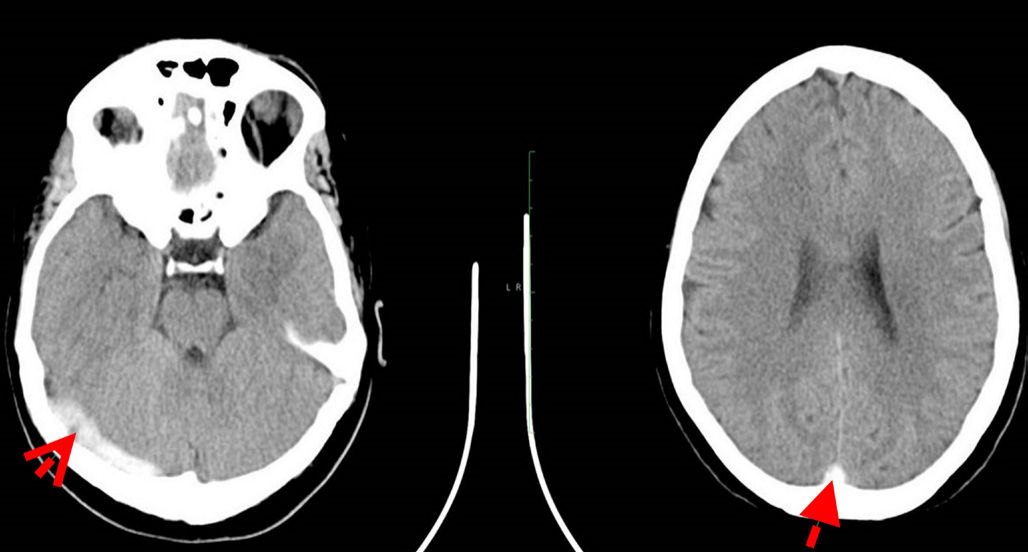
Brain CT findings on admission. The first red arrow shows a left parasagittal parietal lobe high convexity gyral hypodensity (1.8×0.9 cm) region is seen. Appearances may be caused by a venous infarct. The second red arrow shows an abnormal superior sagittal and left sigmoid sinus hyperdensity suspicious for venous sinus thrombosis (empty delta sign). Relative hypodensity in the left internal capsule. No other areas of abnormal attenuation. Otherwise normal appearances of the brain parenchyma, ventricles, cisterns and nuclei. No extra-axial collections. No intraparenchymal haemorrhage detected. Impression: left parietal lobe venous infarct. Venous dural sinus thrombosis.

**Figure 2:**
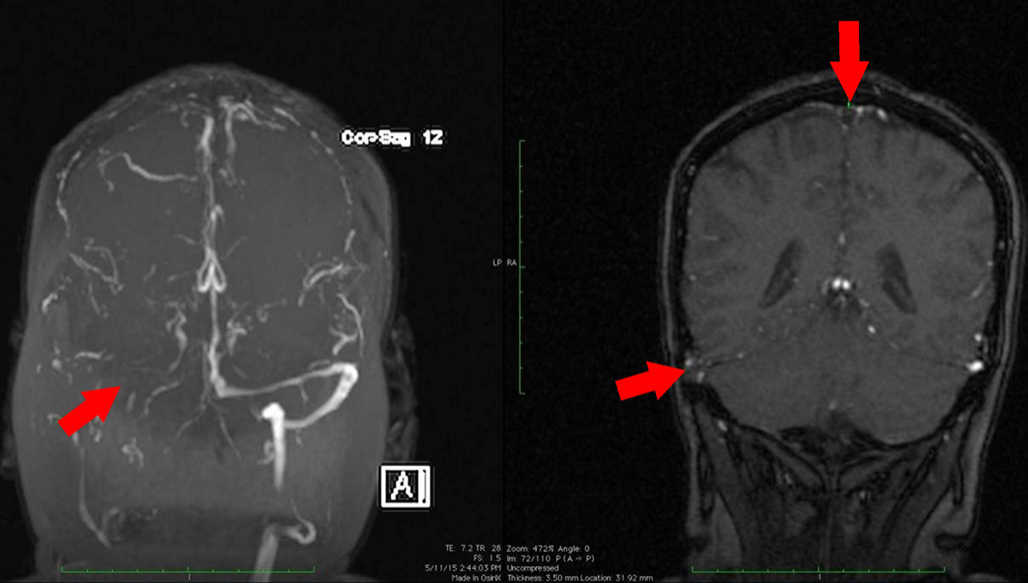
Brain MRI and MRV findings on admission. The red arrows show a signal void seen in the posterior 2/3 of the superior sagittal sinus, as well as within the right transverse and sigmoid sinuses. Correlation with CT suggests venous sinus thrombosis.

All routine blood investigations, including complete blood count, liver function tests, renal function tests, fasting lipids and electrolytes were all within normal limits. Furthermore, prothrombin time, partial thromboplastin time and international normalised ratio (INR) were 11 s, 26.2 s and 1.03, (normal range: 9.5–13.5, 27.0–35.0 and 0.8–1.2 s), respectively, as well as an estimated sedimentary rate of 23 mm/hour (0–15) and C reactive protein was 0.7 mg/dL (0–0.5). Antiphospholipid and thrombophilia screens were normal: Protein C 95.6% (70–140), Protein S 113% (63–135), Factor V Leiden 34.6 s (28–50), antithrombin III 117% (85–125) and anticardiolipin antibodies IgA 2.2 (<12 APL U/mL), IgG <2 (<12 GPL U/mL) and IgM 3.70 (<12 MPLU/mL). Urinalysis was normal. A brain CT scan (figure 1) showed two areas of hypodensity in the left parietal lobe and in the left internal capsule, as well as a region of abnormal hyperdensity in the left sigmoid and superior sagittal sinus. These findings were suspicious of a left parietal lobe venous infarct, secondary to a venous dural sinus thrombosis, with MRI and MRV (figure 2) confirming a signal void seen in the posterior 2/3 of the superior sagittal sinus, as well as within the right transverse and sigmoid sinuses.

A diagnosis of drug (norethisterone enanthate)-induced CVST was considered.

## Treatment

The patient was treated with antioedema measures comprising mannitol 50 mL intravenously two times for 24 hours and dexamethasone 4 mg orally one time for 3 days. Anticoagulation was initiated with warfarin after bridging with low-molecular-weight heparin enoxaparin.

## Outcome and follow-up

The patient recovered completely within 1 week and was discharged. Her condition remained well throughout her follow-up visits. Anticoagulation was continued for 6 months as recommended, though recanalisation is expected after 3 months of anticoagulation therapy.^4^ She was also advised to avoid prothrombotic drugs and to use barrier contraception.

**Figures 3:**
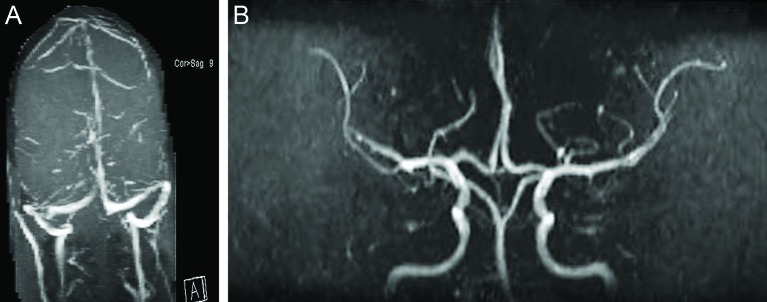
(A and B) Follow-up brain MRI and MRV after 6 months. Normal MRI and MRV of the brain with no defects in the superior sagittal, right transverse and sigmoid sinuses, and no acute intracranial haemorrhages.

A follow-up MRI brain with a venogram (figure 3A,B), performed 6 months after the initial presentation confirmed recanalisation, as it revealed no thrombosis in the superior sagittal, right transverse and sigmoid sinuses, and no acute intracranial haemorrhages. All anticoagulation medications were stopped, and the patient was advised lifelong avoidance of norethisterone enanthate contraception.

## Discussion

CVST is a rare and potentially fatal condition. This patient’s presentation with a severe headache is present in 90% of the cerebral venous thromboses cases; the list of differential diagnoses can be infectious, inflammatory, structural and even psychological disorders.^5^ Furthermore, its occurrence in isolation, in the absence of focal neurological signs or papilloedema, and with normal antiphospholipid and thrombophilia screen, poses an even greater diagnostic challenge. This feature has been shown to occur in only 15% of CVST cases.^6^ A retrospective, cross-sectional study conducted by Coutinho *et al*, analysed adult cerebral thromboses in 19 hospitals located in two Dutch provinces serving 3.1 million, (which is approximately three times the population of Trinidad), between 1 January 2008 and 31 December 2010. The study revealed an overall annual incidence of 1.32 per 100 000, a significantly higher incidence in women than men (1.86 vs 0.75), and a higher incidence among patients aged 31–50 years (1.71) with a median age of 41 years. Additionally, 52% of female patients used oral contraceptives, and 18% were pregnant or had recently given birth.^7^

Diagnosis of CVST is made using contrast-enhanced CT, MRI or MRV. Contrast-enhanced CT shows the classic empty delta sign, as was seen in this case (figure 1). This is present in 10%–35% of cases, and is produced by an intraluminal filling defect surrounded by contrast in the posterior part of the superior sagittal sinus.^8^ The combination of an abnormal signal in a sinus and a corresponding absence of flow on MRV confirms the diagnosis of CSVT.^8^ The American Heart Association/American Stroke Association 2011 Scientific Statement recommends MRI/MRV as the imaging test of choice for evaluation of suspected cerebral venous thrombosis. Angiography (brain) is reserved for situations in which MRV or CT is inconclusive.^9^ Thrombosis has been reported to occur in the superior sagittal, transverse and sigmoid sinuses in 62%, 41%–45% and 10% of cases, respectively.^9^

Thrombosis of the cerebral veins causes local effects by venous obstruction, which results in oedema of the brain and venous infarction. Thrombosis of the major sinuses causes impaired absorption of cerebrospinal fluid (CSF) and intracranial hypertension.^10^ These mechanisms are responsible for the four possible clinical syndromes that are seen. These include: isolated intracranial hypertension, which presents as a severe headache (90%); focal neurological deficits (44%) such as hemiparesis, which usually becomes bilateral in a few days; seizures (30%–40%), which occur more frequently with thrombosis of the sagittal sinus and cortical veins; and encephalopathy (22%), which can result from thrombosis of the straight sinus or from extensive cerebral oedema, large venous infarcts or parenchymal haemorrhages.^11^ At least one risk factor could be identified in more than 85% of patients with CVST.^11^ Coutinho *et al* analysed data from the International Study on Cerebral Vein and Dural sinus Thrombosis (ISCVT), a multicentre prospective observational study, and found that 465 of the 624 patients were women (75%), and a gender-specific risk factor (oral contraceptives, 46%; pregnancy or puerperium, 17%; hormonal replacement therapy, 3%) was present in 65% of the women. Women had a better prognosis than men (complete recovery 81% vs 71%). Congenital and acquired thrombophilia carried similar percentages for men and women (25% vs 22% and 15% vs 16%, respectively). Of note, the incidence of infection and malignancy, as causes of CVST, were two times higher in men than in women (21% vs 10% and 11% vs 6%, respectively).^12^

The patient’s blood and urine investigations were within normal limits. Dehydration was excluded in view of her moist mucous membranes, normal skin turgor and renal function tests. She had no predisposing risk factors associated with cerebral vein thrombosis, except for the use of the progestin-only contraceptive injection.

Many observational studies have shown that combined oral contraceptives are associated with a twofold to sixfold increased risk of venous thrombosis.^13–15^ The oestrogen compound (ethinyloestradiol) in oral contraceptive formulations is thought to cause the increased risk of thrombosis, as a reduction in the dose of this compound resulted in a reduced risk of venous thrombosis. Oral contraceptives induced a degree of activated protein C resistance comparable with the resistance caused by a factor V Leiden mutation.^16^ Clinical studies have shown that this effect on coagulation factors was more pronounced in oral contraceptives containing desogestrel (a third-generation progestogen) than in levonorgestrel (a second-generation progestogen), which may be explained by a less effective compensation of the thrombotic effect of ethinyloestradiol by desogestrel.^17^ Thus, factors associated with an increased risk of venous thrombosis are higher doses of ethinyloestradiol, as well as a third-generation progestogen.^18^ However, these findings cannot be related with the depot injections of norethisterone enanthate which our patient used.

A study performed by McEwan *et al* monitored 56 women using depot-norethisterone enanthate injections for 2 years and compared them to a control group of 48, and found there were no significant differences between the treatment and control groups with respect to factor VIIc and antithrombin. Factor Xc, however, was reduced in women who had taken the injections for over 2 years, but less than 5 years; while haemoglobin levels, red cell count and packed cell volume were higher in the treatment group as compared with those in the control group. Therefore, this study concluded that long-term use of norethisterone enanthate is not associated with any markedly deleterious effects on factors VIIc and Xc, antithrombin III or haemoglobin levels.^19^ The drug norethisterone enanthate, a first-generation progestogen, has a lower risk of causing venous thrombosis when compared with third-generation progestogens. It has been shown to be partly metabolised to ethinyloestradiol, which has been associated with an increased risk of venous thrombosis.^18 20^

The main priority in the treatment for CVST is stabilisation, prevention or reversal of cerebral oedema, and herniation with intravenous mannitol, (which was used in our patient) or decompressive hemicraniectomy, and/or removal of haemorrhagic infarct with surgical procedures.^21^ A prospective study published in the ISCVT showed that 79% of patients recovered from this treatment.^21^ Although there is a risk of venous infarcts becoming haemorrhagic, anticoagulation therapy still forms the mainstay of treatment.^22^ Our patient showed full clinical and radiological improvement following administration of low-molecular-weight heparin and mannitol. Anticoagulation (warfarin) with a target INR of 2.5 was continued for 6 months as there were no predisposing conditions.^23^ No further treatment was advised, except the avoidance of norethisterone enanthate depot injections.

The prognosis of CVST is usually favourable, with more than 80% of patients, as in our case, having a good neurological outcome.^24^

**Learning points**

- Cerebral venous sinus thrombosis (CVST) is a rare condition, and its presentation can mimic various benign conditions.
- Clinicians should have a high level of suspicion, identifying aetiology and predisposing conditions, to facilitate a prompt diagnosis.
- More research is needed to confirm or exclude a causal link between norethisterone enanthate and CVST.
- CVST management should be aggressive to ensure the best possible outcome for patients.
- Prognosis for CVST is usually favourable.
